# Sustainability indicators for bioenergy generation from Amazon׳s non-woody native biomass sources

**DOI:** 10.1016/j.dib.2018.11.022

**Published:** 2018-11-14

**Authors:** Josmar Almeida Flores, Odorico Konrad, Cíntia Rosina Flores, Nádia Teresinha Schroder

**Affiliations:** a Graduate Program in Environment and Development, Universidade do Vale do Taquari, Lajeado, Rio Grande do Sul, Brazil; b Universidade Federal de Rondônia, Porto Velho, Rondônia, Brazil; c Graduate Program in Health Promotion, Human Development, and Society, Universidade Luterana do Brasil, Canoas, Rio Grande do Sul, Brazil

**Keywords:** Brazilian Amazon, Bioenergy, Indicators, Non-wood biomass, Sustainability

## Abstract

This data article focuses on sustainability indicators for bioenergy generation from Brazilian Amazon׳s non-woody native biomass sources, considered to be modern forms of biomass. In the construction of the indicators, the Indicator-based Framework for Evaluation of Natural Resource Management Systems (MESMIS, from the original Spanish) method was used, with the application of the seven sustainability attributes to identify critical points and limiting and favorable factors for sustainability. The data yielded a list of 29 indicators distributed across 27 critical points, selected from three system evaluation areas: 11 environmental indicators, 11 social indicators, and 7 economic indicators.

## Graphical abstract

**Figure:**
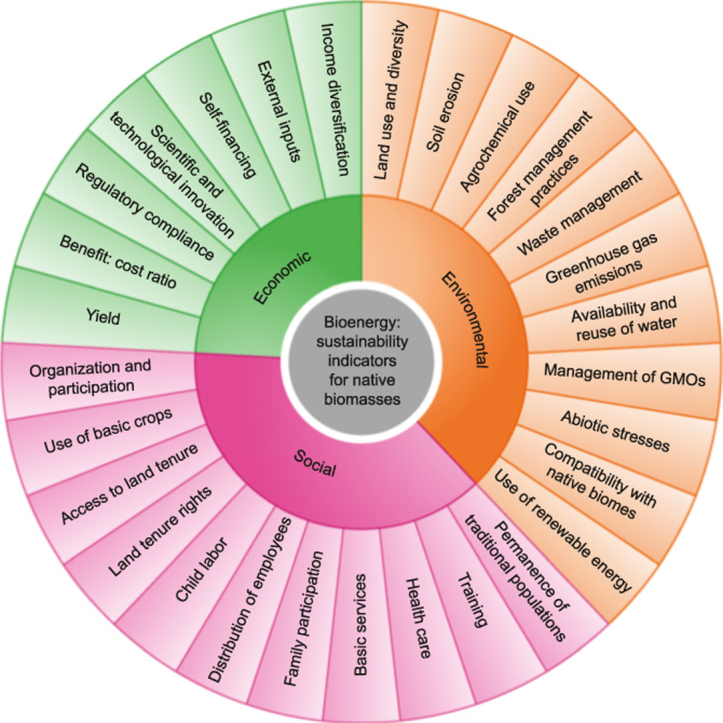

**Specifications table**

**Table:** 

Subject area	*Environmental science*
More specific subject area	*Sustainability*
Type of data	*Tables, text file*
How data were acquired	*Literature review*
Data format	*Filtered and analyzed*
Experimental factors	*In the construction of the sustainability indicators, the Indicator-based Framework for Evaluation of Natural Resource Management Systems (MESMIS, from the original Spanish ‘Marco para la Evaluación de Sistemas de Manejo de Recursos Naturales Incorporando Indicadores de Sustentabilidad’) method was used, considering the seven sustainability attributes to identify critical points and their respective indicators.*
Experimental features	*Data are focused on the development of sustainability indicators to be used in bioenergy production systems from Brazilian Amazon׳s non-woody native biomass sources*
Data source location	*Brazilian Amazon*
Data accessibility	*Data included with this article*
Related research article	*None*

**Value of the data**

• The data contribute to minimizing the impact of non-renewable energy sources by expanding research into modern biomass for bioenergy generation.
• Sustainability indicators are needed to evaluate the suitability of Amazon׳s non-woody native biomass an alternative source for bioenergy production.
• The data describe sustainability indicators used in the Brazilian and international literature for bioenergy.
• Adequate application of these data may fill the gaps of sustainability evaluation of Amazon׳s non-woody native biomass sources for bioenergy generation, a still incipient area of knowledge.
• The data provide information on bioenergy sustainability indicators that can be used for decision-making.

## 1 Data

This data article focuses on sustainability indicators for bioenergy production systems that use Brazilian Amazon׳s non-woody native biomass sources. The creation of this data set is based on the analysis of globally recognized scientific certifications and publications related to sustainability standards for the biomass-bioenergy sector. The data of the sample were processed and shared in the Supplementary material of this data article as a Microsoft Excel spreadsheet (XLSX file) containing raw extracted and filtered data.

The data yielded a list of 29 sustainability indicators for bioenergy generation from Amazon׳s biomass native sources, selected from seven sustainability attributes: productivity, stability, reliability, resilience, adaptability, equity, and self-reliance. Table 1 shows the 27 critical points identified for the bioenergy production system analyzed in this study, linked to their respective sustainability attributes. Table 2 then describes the indicators linked to these critical points, according to the system evaluation area.

**Table 1:** Critical points identified for bioenergy production systems that use Amazon׳s non-woody native biomass sources.

Sustainability attributes	Critical points	References
Productivity	1. Productivity	[1], [2], [3]
	2. Profitability	[1], [2], [3]
	3. Regulatory compliance	[1], [2], [4]
	4. Soil degradation	[1], [2], [3], [4]
	5. Pollution	[1], [4]
Stability, reliability, resilience	6. Food competition	[1], [2], [3], [4]
	7. Use of forest management practices	[1], [4]
	8. Waste disposal	[1], [2]
	9. Greenhouse gas emissions	[1], [2], [3], [4]
	10. Availability and reuse of water	[1], [2], [3]
	11. Use of genetically modified organisms (GMOs)	[1]
	12. Vulnerability to external effects	[1]
	13. Biodiversity and ecosystems	[2], [4]
	14. Desertion of the area	[1], [3], [4]
Adaptability	15. Technological innovation	[1], [4]
	16. Capacity	[1], [2], [3], [4]
Equity	17. Basic services	[1], [2], [3], [4]
	18. Family involvement	[1], [3], [4]
	19. Equal opportunities	[1], [2], [3], [4]
	20. Child labor	[1], [2], [3], [4]
	21. Land rights	[1], [2], [3], [4]
	22. Food competition	[1], [2], [3], [4]
Self-reliance	23. Dependence on subsidies	[1], [2], [4]
	24. Dependence on external inputs	[1]
	25. Dependence on fossil fuels	[1], [3], [4]
	26. Sources of income	[1], [3], [4]
	27. Organization and participation	[1], [4]

**Table 2:** Sustainability indicators by critical point and evaluation area.

Evaluation area	Indicators	Critical points	References
Environmental	1. Land use and diversity	Food competition	[1], [2], [3], [4]
	2. Soil erosion	Soil degradation	[1], [2], [3], [4]
	3. Agrochemical use	Pollution	[1], [4]
	4. Forest management practices	Use of forest management practices	[1], [4]
	5. Waste management	Waste disposal	[1], [2]
	6. Greenhouse gas emissions	Greenhouse gas emissions	[1], [2], [3], [4]
	7. Availability and reuse of water	Availability and reuse of water	[1], [2], [3]
	8. Management of GMOs	Use of GMOs	[1]
	9. Abiotic stresses	Vulnerability to external effects	[1]
	10. Compatibility with native biomes	Biodiversity and ecosystems	[2], [4]
	11. Use of renewable energy	Dependence on fossil fuels	[1], [3], [4]
Social	12. Permanence of traditional populations	Desertion of the area	[1], [3], [4]
	13. Training	Capacity	[1], [2], [3], [4]
	14. Health care	Basic services	[1], [2], [3], [4]
	15. Basic services	Basic services	[1], [2], [3], [4]
	16. Family participation	Family involvement	[1], [3], [4]
	17. Distribution of employees	Equal opportunities	[1], [2], [3], [4]
	18. Child labor	Child labor	[1], [2], [3], [4]
	19. Land tenure rights	Land rights	[1], [2], [3], [4]
	20. Access to land tenure	Land rights	[1], [2], [3], [4]
	21. Use of basic crops	Food competition	[1], [3], [4]
	22. Organization and participation	Organization and participation	[1], [4]
Economic	23. Yield	Productivity	[1], [2], [3]
	24. Benefit-cost ratio	Profitability	[1], [2], [3]
	25. Regulatory compliance	Regulatory compliance	[1], [2], [4]
	26. Scientific and technological innovation	Technological innovation	[1], [4]
	27. Self-financing	Dependence on subsidies	[1], [2], [4]
	28. External inputs	Dependence on external inputs	[1]
	29. Income diversification	Sources of income	[1], [3], [4]

**Fig. 1:**
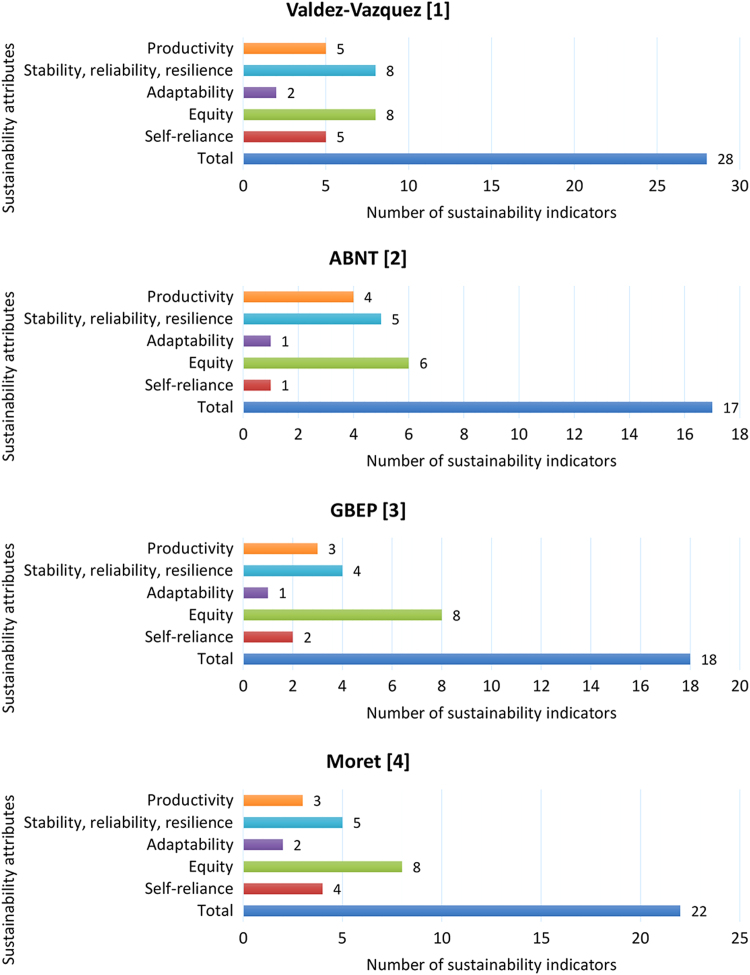
Number of indicators per sustainability attributes coded by a sample model.

**Fig. 2:**
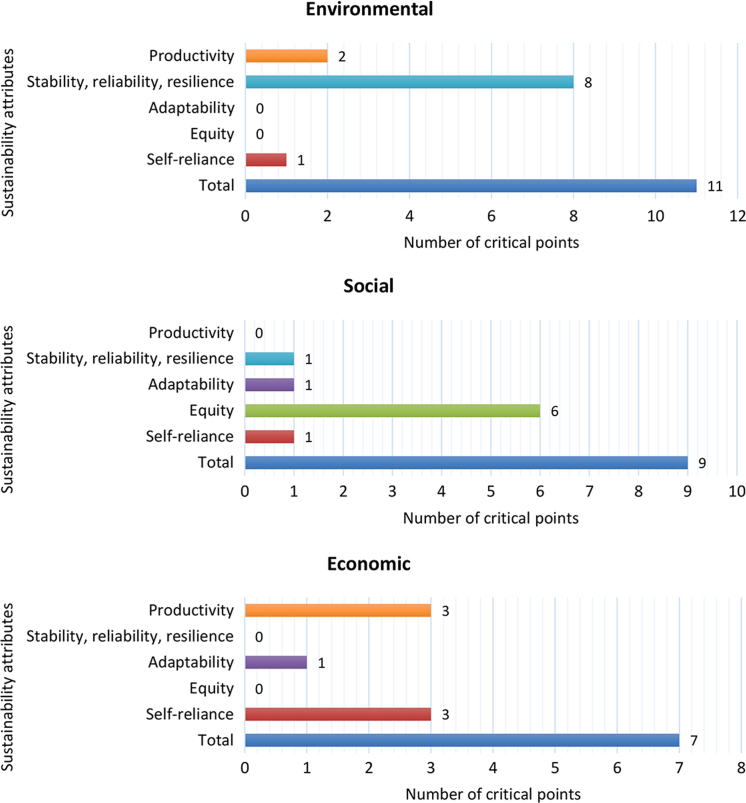
Number of critical points per sustainability attributes coded by evaluation area.

Based on the data set of the sample, it was possible to categorize and quantify the indicators and critical points. This stratification was performed according to the respective evaluation area, for each one of the seven sustainability attributes (Table 3). Figs. 1 and 2 provide a comprehensive visual comparison of sustainability attributes in relation to indicator and critical points standards for bioenergy production systems that use Brazilian Amazon׳s non-woody native biomass sources.

**Table 3:** Number of indicators and critical points per sustainability attributes.

Sustainability attributes	Critical points by evaluation area				Indicators by sample			
	Environmental	Social	Economic	Total	Valdez-Vazquez [1]	ABNT [2]	GBEP [3]	Moret [4]
Productivity	2	–	3	5	5	4	3	3
Stability, reliability, resilience	8	1	–	9	8	5	4	5
Adaptability	–	1	1	2	2	1	1	2
Equity	–	6	–	6	8	6	8	8
Self-reliance	1	1	3	5	5	1	2	4
Total	11	9	7	27	28	17	18	22

## 2 Experimental design, materials, and methods

### 2.1 Study area description

**Fig. 3:**
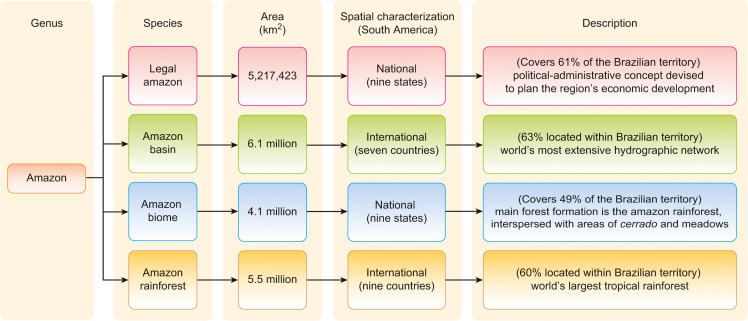
Explaining the various Amazons.

In the construction of the sustainability indicators of non-woody native biomass, the study area was the Amazon biome, which integrates the various Amazons. Fig. 3 shows that the term “Amazon” is used in several different ways at the global and regional levels, and, although these are interrelated, they have distinct meanings [5].

### 2.2 System characterization: description of the study biomass sample

**Fig. 4:**
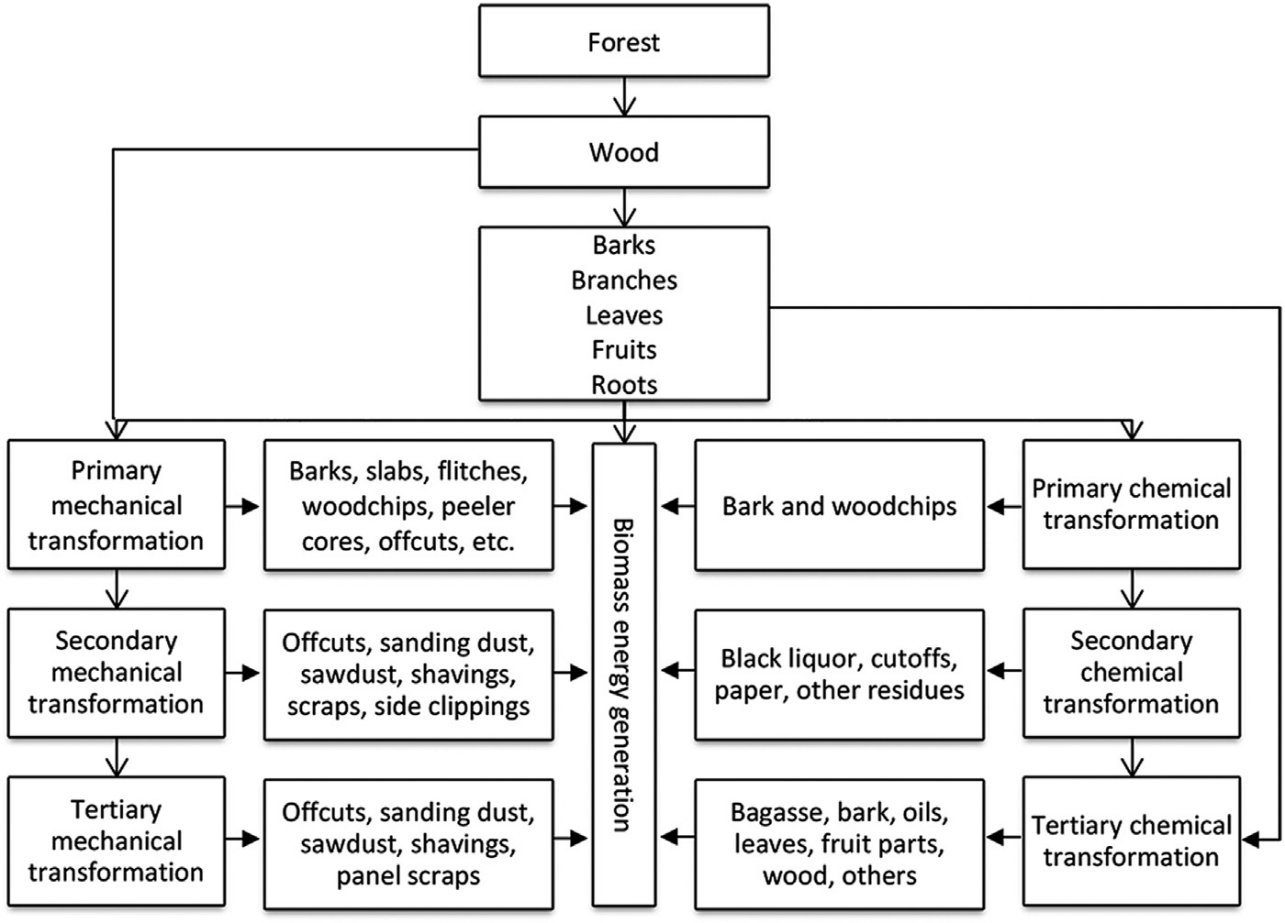
Physical flow of forest biomass for energy generation. Source – Brand [7].

Considering the universe of forest biomass, the construction of the indicators aimed the creation of sustainability parameters for Amazon׳s non-woody native biomass sources (fruits) [6], which are modern forms of biomass. Selection followed the typology described by Brand [7] in the physical flow of forest biomass for energy generation (Fig. 4).

### 2.3 Construction of sustainability indicators

The process of construction of sustainability indicators of non-woody native biomass sources for bioenergy generation relies on the Indicator-based Framework for Evaluation of Natural Resource Management Systems method (MESMIS, from the Spanish Marco para la Evaluación de Sistemas de Manejo de Recursos Naturales Incorporando Indicadores de Sustentabilidad). MESMIS is aimed at researchers and professionals from different areas of knowledge who are interested in developing and disseminating tools for sustainability evaluation systems. It can be applied in case studies in the rural sector, especially in the rural context of Latin America [8].

**Fig. 5:**
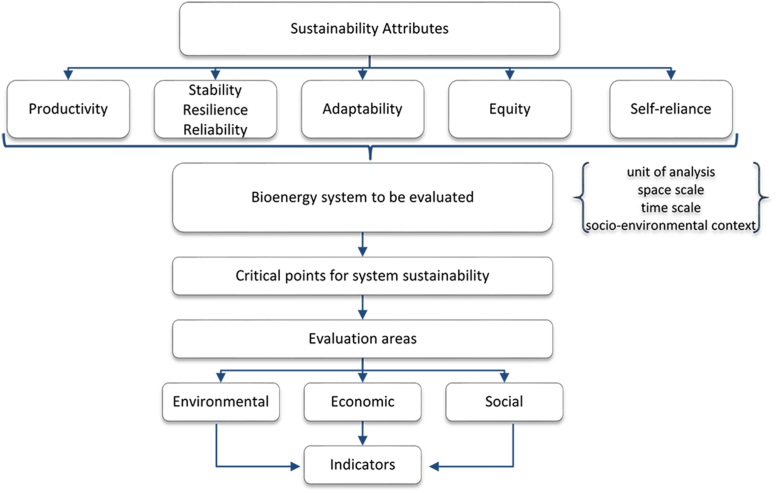
Graphical representation of the MESMIS method for evaluation of bioenergy from Amazon׳s non-woody native biomass sources.

MESMIS is characterized by its flexibility and adaptability to different levels of information and technical training, providing a participatory and interdisciplinary approach that allows the adaptation of the sustainability evaluation process to the specificities of each study [9], [10], [11]. This flexibility was used for the identification of critical points, determination of diagnostic criteria, and definition of sustainability indicators (Fig. 5) [12].

As described in the MESMIS method, the seven sustainability attributes (productivity, stability, reliability, resilience, adaptability, equity, and self-reliance) used for identification of critical points were applied to the present system, revealing limiting and favorable factors for sustainability.

For selection of critical points and subsequent sustainability evaluation for bioenergy, different types of scientific publications were used. Table 4 presents the composition of the sample, characterized by documents that varied in terms of geographical scope, authorship, and typology. The diversity of these publications allowed the data to encompass multiple viewpoints and goals of sustainability indicators for bioenergy. Then, each of the selected critical points was linked to sustainability indicators structured into the economic, environmental, and social dimensions.

**Table 4:** Composition of the sample applied to the sustainability attributes.

Document	Origin	Typology	Scope	Date of publication
ABNT ISO 13065: Sustainability criteria for bioenergy [2]	International Organization for Standardization	Technical standard	International	2015

Description: This standard specifies principles, criteria and indicators for the bioenergy supply chain to facilitate evaluation of environmental, social and economic aspects of sustainability.				
Sustainability criteria and indicators for bioenergy [4]	Working Group on Energy of the Brazilian Forum of NGOs and Social Movements for the Environment and Development	Technical paper	National	2006

Description: Set of sustainability criteria and indicators to guide discussion among the various social and economic segments involved in enterprises of energy generation from biomass, in its social, environmental, and economic dimensions				
Proposal for a sustainability evaluation framework for bioenergy production systems using the MESMIS methodology [1]	Renewable and Sustainable Energy Reviews	Scientific article	International	2017

Description: The aim of the present study is to develop a sustainability evaluation framework that is suitable to Bioenergy Production Systems, integrating any feedstock, technological process, and social component for low and middle-income countries.				
Sustainability Indicators for Bioenergy [3]	Global Bioenergy Partnership (GBEP)	Technical paper	International	2011

Description: This report presents 24 indicators of sustainability regarding the production and use of modern bioenergy, broadly defined. The indicators were developed by the Global Bioenergy Partnership (GBEP) and provide a framework for assessing the relationship between production and use of modern bioenergy and sustainable development. The indicators were intentionally crafted to report on the environmental, social, and economic aspects of sustainable development.				

The sample allowed the data set to encompass the characteristics of representativeness, comparability, clarity and synthesis, data collection, and forecasting and goals [13].

## References

[1] Valdez-Vazquez I., Gastelum C.R.S., Escalante A.E. (2017). Proposal for a sustainability evaluation framework for bioenergy production systems using the MESMIS methodology. Renew. Sustain. Energy Rev..

[2] Associação Brasileira de Normas Técnicas (ABNT). ISO 13065/2015 Norma Traduzida: critérios de sustentabilidade em bioenergia, ABNT, Rio de Janeiro, 2016.

[3] Global Bioenergy Partnership (GBEP) (2011). The Global Bioenergy Partnership Sustainability Indicators for Bioenergy.

[4] A. Moret, D. Rodrigues, L. Ortiz, Critérios e indicadores de sustentabilidade para bioenergia, GT Energia do Fórum Brasileiro de ONGs e Movimentos Sociais (FBOMS). 2006.

[5] E. Silva, J.A.G. Pereira, A nova Economia da floresta. 390 (2018) 24–35.

[6] Flores J.A., Konrad O., Flores C.R., Schroder N.T. (2018). Inventory data on Brazilian Amazon׳s non-wood native biomass sources for bioenergy production. Data Brief.

[7] M.A. Brand, Energia de biomassa florestal, Interciência, Rio de Janeiro, 2010.

[8] Marco para la evaluación de sistemas de manejo de recursos naturales incorporando indicadores de sustentabilidade (MESMIS), El proyecto mesmis, 2018. 〈http://www.mesmis.unam.mx:8080/MESMIS2/〉. (Accessed 31 May 2018).

[9] Acosta-Alba I., Van der Werf H.M.G. (2011). The use of reference values in indicator-based methods for the environmental assessment of agricultural systems. Sustainability.

[10] M. Astier, O. Masera, Y. Galván-Miyoshi, Evaluación de Sustentabilidad: un enfoque dinámico y multidimensional, SEAE/CIGA/ECOSUR/CIEco/UNAM/GIRA/Mundiprensa/FundaciónInstituto de Agricultura Ecológica y Sustentable, Espanha, 2008.

[11] Verona L.A.F. (2010). A real sustentabilidade dos modelos de produção da agricultura: indicadores de sustentabilidade na agricultura. Hortic. Bras..

[12] Cândido G.A., Nóbrega M.M., Figueiredo M.T.M., Maior M.M.S. (2015). Sustainability assessment of agroecological production units: a comparative study of idea and mesmis methods. Ambient. Soc..

[13] Bianchi A.L., Lima A.A.M., Dias S.S., Philippi Jr. A., Reis L.B. (2016). Indicadores energéticos e sustentabilidade. Energia e sustentabilidade.

